# Commentary: Creativity and Memory: Effects of an Episodic-Specificity Induction on Divergent Thinking

**DOI:** 10.3389/fpsyg.2016.00824

**Published:** 2016-05-27

**Authors:** Anna Abraham

**Affiliations:** School of Social Sciences, Leeds Beckett UniversityLeeds, UK

**Keywords:** creativity, episodic memory, imagination, declarative memory, divergent thinking

Madore et al. (2015) reported evidence in support of the idea that episodic-specificity induction facilitates creative thinking. In this Commentary, I draw attention to the issue of clarity in the targeting of creativity-relevant operations. The basis of these concerns is grounded in the atypical nature of the adopted methodological protocols as well as the terminology used to refer to the measures of interest. Such inconsistencies could lead to confusion or the perpetuation of misguided notions. The objective of this commentary is to therefore lay bare these concerns so that future investigations based on this study will be mindful of the same.

The information processing mechanisms underlying creativity have for long been discussed in relation to operations of declarative memory such that individual differences in creativity are held to emerge from variability in the manner in which stored concepts are either accessed from or associated with one another within long-term semantic memory networks (Mednick, 1962; Mendelsohn, 1974). Although, explicit formulations on how the processes that underlie creative thought tie together with other aspects of imagination are fairly recent (Abraham and Bubic, 2015; Beaty et al., 2016), the idea that free-associative episodic thinking is intertwined with creative operations was, in fact, highlighted in one of the first neuroimaging studies of episodic memory (Andreasen et al., 1995). The novel finding of the selective impact of inducing episodic retrieval strategies on idea generation therefore represents a timely development (Madore et al., 2015). What needs to be clarified is which aspects of creativity are influenced by episodic-specific induction as this is not readily apparent from the paper.

The main concern stems from the unorthodox scoring protocol adopted by Madore et al. (2015) for the Alternate Uses Task (AUT), where participants generate as many uses as possible for common objects. Four measures are typically derived from the AUT: fluency (number of discrete uses), originality (degree of unusualness of the uses), flexibility (number of discrete categories of uses), and elaboration (the degree of detail associated with the uses). Which of these are assessed depends on the aims of the study in question and the version of the AUT being adopted (Guilford et al., 1960; Wallach and Kogan, 1965). In the manifold studies that have used the AUT, appropriateness is an inclusion criterion for determining a use to be valid. In fact, the AUT manual clearly states, “A use, to be acceptable, should be possible for the object” (Guilford et al., 1960, p. 30). So an inappropriate use is not evaluated further when deriving the AUT measures. This is where the Madore et al. (2015) protocol departs sharply from the standard as they evaluate all uses when determining some measures. In line with another study that has used a similar, but not identical, protocol (Addis et al., 2016), uses deemed appropriate received a score of 1 and inappropriate uses a score of 0. The only AUT measures in relation to which they report significant findings across both experiments as a function of induction type (as reflected by significant interaction effects: induction × task) were termed “categories of appropriateness” (in the main article) and “appropriateness” (in the Supplemental Material).

The potential for considerable confusion enters here because the “categories of appropriateness” measure reflects the number of categories of appropriate uses, which corresponds to the standard AUT “flexibility” measure. The same is true of “appropriateness,” which reflects the number of acceptable uses, as this corresponds to the standard AUT “fluency” measure. The authors adopt a different notion of flexibility and fluency as also incorporating inappropriate uses (Supplemental Material). No grounds have been forwarded to explain the necessity for such differences from the standard protocol^1^ nor have they been explicitly acknowledged in the paper.

It is worth noting that the authors also assessed the degree of overall creativity associated with the generated uses. Here again, the AUT “originality” measure was not referred to but a similar “creativity” measure^2^ was derived following another scoring protocol (Benedek et al., 2014) which reflects “how original and unusual each use was.” Across both experiments, episodic-specificity induction was not found to have a significant impact on this measure, nor on the elaboration measure.

So the takeaway message in terms of divergent thinking is that episodic-specificity induction has a significant impact on fluency and flexibility, but does not have a significant impact on originality and elaboration.

Using standard and clear terminology serves as a great aid in being able to relate exciting novel findings to the published literature. For instance, episodic based strategies are dominantly used in the early phase of idea generation and are associated with the generation of already known—and therefore personally unoriginal—uses (Gilhooly et al., 2007). Episodic-specificity induction is linked to an increase in the number of generated details in memory and imagination (Madore and Schacter, 2016), but only the AUT elaboration score is positively correlated with the level of internal detail given during episodic simulation of both past and future events (Addis et al., 2016). It would be useful to explore how different components of episodic cognition tie in with select facets of creativity.

It is crucial that a topic as vital as the study of creativity rapidly become less of a niche domain in cognitive psychology and neuroscience, and that more researchers the world over invest their expertise, energy, and indeed creativity to explore this singularly rich and central facet of human life. The Madore et al. (2015) paper provides the right kind of impetus for this to happen. It is essential though that clarity and specificity at the level of definition be maintained as this will serve to facilitate the extent to which findings based on episodic memory approaches will be accurately embedded within the larger literature on creativity.

## Author contributions

The author confirms being the sole contributor of this work and approved it for publication.

### Conflict of interest statement

The author declares that the research was conducted in the absence of any commercial or financial relationships that could be construed as a potential conflict of interest.

## Footnotes

^1^Similar inconsistencies also apply to the control object association task, where participants are given an object cue and listed other objects that are typically associated with it. However, the study cited as the source for this task (Abraham et al., 2012) employed the object location task, where participants received a location cue and were required to recall objects that would be typically found in that location. These differences are not inconsequential as associative and scene construction processes would be differentially engaged by both tasks.

^2^ The authors stated that this measure and the elaboration measure was focused on in relation to the “categories of appropriate uses” measure, but what this means is unclear.

## References

[B1] Abraham A. Bubic A. (2015). Semantic memory as the root of imagination. Cogn. Sci. 6:325. 10.3389/fpsyg.2015.00325 25852626 PMC4371585

[B2] Abraham A. Pieritz K. Thybusch K. Rutter B. Kröger S. Schweckendiek J.. (2012). Creativity and the brain: uncovering the neural signature of conceptual expansion. Neuropsychologia 50, 1906–1917. 10.1016/j.neuropsychologia.2012.04.015 22564480

[B3] Addis D. R. Pan L. Musicaro R. Schacter D. L. (2016). Divergent thinking and constructing episodic simulations. Memory 24, 89–97. 10.1080/09658211.2014.985591 25483132 PMC4458228

[B4] Andreasen N. C. O'Leary D. S. Cizadlo T. Arndt S. Rezai K. Watkins G. L.. (1995). Remembering the past: two facets of episodic memory explored with positron emission tomography. Am. J. Psychiatry 152, 1576–1585. 7485619 10.1176/ajp.152.11.1576

[B5] Beaty R. E. Benedek M. Silvia P. J. Schacter D. L. (2016). Creative cognition and brain network dynamics. Trends Cogn. Sci. 20, 87–95. 10.1016/j.tics.2015.10.004 26553223 PMC4724474

[B6] Benedek M. Jauk E. Fink A. Koschutnig K. Reishofer G. Ebner F.. (2014). To create or to recall? Neural mechanisms underlying the generation of creative new ideas. NeuroImage 88, 125–133. 10.1016/j.neuroimage.2013.11.021 24269573 PMC3991848

[B7] Gilhooly K. J. Fioratou E. Anthony S. H. Wynn V. (2007). Divergent thinking: strategies and executive involvement in generating novel uses for familiar objects. Br. J. Psychol. 98(Pt 4), 611–625. 10.1348/096317907X173421 17535464

[B8] Guilford J. P. Christensen P. R. Merrifield P. R. Wilson R. C. (1960). Alternate Uses Manual. Menlo Park, CA: Mind Garden.

[B9] Madore K. P. Addis D. R. Schacter D. L. (2015). Creativity and memory: effects of an episodic-specificity induction on divergent thinking. Psychol. Sci. 26, 1461–1468. 10.1177/0956797615591863 26205963 PMC4567456

[B10] Madore K. P. Schacter D. L. (2016). Remembering the past and imagining the future: Selective effects of an episodic specificity induction on detail generation. Q. J. Exp. Psychol. (Hove). 69, 285–298. 10.1080/17470218.2014.999097 25529786 PMC4545482

[B11] Mednick S. A. (1962). The associative basis of the creative process. Psychol. Rev. 69, 220–232. 14472013 10.1037/h0048850

[B12] Mendelsohn G. A. (1974). Associative and attentional processes in creative performance. J. Pers. 44, 341–369.

[B13] Wallach M. A. Kogan N. (1965). Modes of Thinking in Young Children : A Study of the Creativity-Intelligence Distinction. New York, NY: Holt, Rinehart & Winston.

